# Genetic Deficiencies of Hyaluronan Degradation

**DOI:** 10.3390/cells13141203

**Published:** 2024-07-16

**Authors:** Stephen P. Fink, Barbara Triggs-Raine

**Affiliations:** 1 Case Comprehensive Cancer Center, Case Western Reserve University, Cleveland, OH 44106, USA; stephen.fink@case.edu; 2 Department of Biochemistry & Medical Genetics, University of Manitoba, Winnipeg, MB R3E 0J9, Canada; 3 Children’s Hospital Research Institute of Manitoba, Winnipeg, MB R3E 3P4, Canada

**Keywords:** hyaluronidase, HYAL1, HYAL2, HYAL3, SPAM1, CEMIP, CEMIP2, genetic disorder, hyaluronan

## Abstract

Hyaluronan (HA) is a large polysaccharide that is broadly distributed and highly abundant in the soft connective tissues and embryos of vertebrates. The constitutive turnover of HA is very high, estimated at 5 g per day in an average (70 kg) adult human, but HA turnover must also be tightly regulated in some processes. Six genes encoding homologues to bee venom hyaluronidase (*HYAL1*, *HYAL2*, *HYAL3*, *HYAL4*, *HYAL6P/HYALP1*, *SPAM1/PH20*), as well as genes encoding two unrelated G8-domain-containing proteins demonstrated to be involved in HA degradation (*CEMIP/KIAA1199*, *CEMIP2/TMEM2*), have been identified in humans. Of these, only deficiencies in *HYAL1*, *HYAL2*, *HYAL3* and *CEMIP* have been identified as the cause or putative cause of human genetic disorders. The phenotypes of these disorders have been vital in determining the biological roles of these enzymes but there is much that is still not understood. Deficiencies in these HA-degrading proteins have been created in mice and/or other model organisms where phenotypes could be analyzed and probed to expand our understanding of HA degradation and function. This review will describe what has been found in human and animal models of hyaluronidase deficiency and discuss how this has advanced our understanding of HA’s role in health and disease.

## 1. Introduction

Hyaluronan (HA) is a large unbranched sugar polymer and a member of the glycosaminoglycan family (reviewed in [1]). It is comprised of repeating disaccharide units of N-acetyl-glucosamine and D-glucuronic acid linked by alternating β1,3 and β1,4 glycosidic bonds (GlcNAcβ4GlcAβ3)_n_. Although HA’s sugar composition defines it as a member of the glycosaminoglycan family, it differs from other members in that it is larger in size, not sulfated or epimerized, and not attached to a protein during synthesis. Further, in contrast to most glycosaminoglycans whose synthesis takes place in the endoplasmic reticulum, HA is synthesized at the cell surface by HA synthases and extruded into the extracellular space [2,3].

HA is ubiquitously present in vertebrates, but its levels and size vary among tissues. It is most abundant in soft connective tissues such as skin, synovial fluid, the vitreous body and Wharton’s jelly of the umbilical cord [4], as well as in the developing embryo [5]. Elegant in vivo studies have demonstrated that HA is rapidly turned over in mammalian tissues (reviewed in [6,7,8]) with approximately 5 g per day turned over in a 70 kg adult human. Degradation can take place in the tissue where it is synthesized, but much of the HA degradation takes place in local lymph nodes [7,9], and the remaining HA enters the blood where it is removed by the blood filtering organs [10]. HA’s high rate of turnover, combined with its ubiquitous presence in tissues, suggested that a deficiency in HA degradation could have pathological consequences [11]. Indeed, deficiencies in enzymes that degrade less-abundant members of the glycosaminoglycan family result in severe lysosomal storage disorders collectively known as the mucopolysaccharidoses (MPSs, reviewed in [12]. 

**Figure 1:**
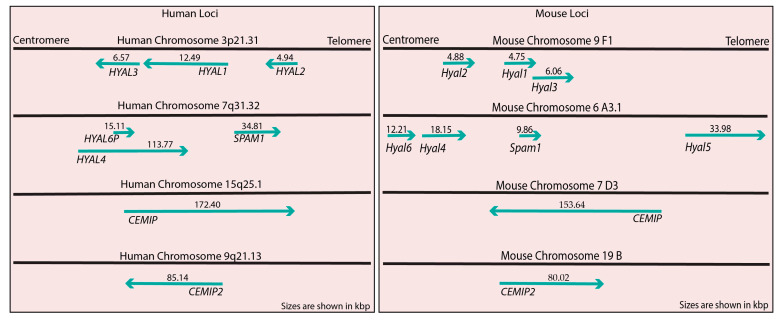
Organization of genes encoding HA degrading proteins in humans and mice. The organization and sizes of the human and mouse genes are based on assemblies NC_000003.12 and NC_000007.14 (human) or NC_00075.7 and NC_000072.7 (mouse). The sizes of the genes are shown above the gene in kilobase pairs. The scaling is unique for each map to allow all hyaluronidase genes in a chromosomal region to be displayed.

The cloning and sequencing of the cDNA encoding bee venom hyaluronidase set the stage for homology-based identification of additional hyaluronidases. These hyaluronidases (EC 3.2.1.35) are endo-β-N-acetyl-hexosaminidases that belong to the glycosidase family 56 which use a substrate-assisted mechanism to cleave the β1-4 glycosidic linkages in HA and, to a lesser extent, chondroitin sulfate [13,14]. PH20 (SPAM1) was the first mammalian hyaluronidase identified based on similarity to the bee venom enzyme [15]. HYAL1 [16], and soon four additional sequence paralogues, were identified in the human genome [17,18,19]. As shown in Figure 1, the genes cluster in two groups of three, *HYAL2*, *HYAL1*, and *HYAL3* on human chromosome 3p21.3 (*Hyal2*, *Hyal1*, and *Hyal3* on mouse chromosome 9F1-F2), and *HYAL4*, *HYAL6P*, and *SPAM1*(*PH20*) on human chromosome 7q31.3 (*Hyal4*, *Hyal6*, and *Spam1/Ph20* on mouse chromosome 6A2) [17,20]. Note that human *HYAL6P* is an expressed pseudogene, whereas in mice it is called *Hyal6* and encodes a protein product. One additional homologue, *Hyal5*, was identified adjacent to the *Hyal4*, *Hyal6*, and *Spam1/Ph20* cluster in rodents [21]. These genes are all believed to have evolved from a common ancestral chondroitinase through gene duplication to form a three-gene cluster followed by a complete duplication of the cluster (reviewed in [22]). The presence of *Hyal5* only in rodents is an indicator of ongoing evolution.

The human hyaluronidase genes on chromosome 3p21.3 were first identified in a 630 kbp interval that was frequently deleted in *LU*ng *Ca*ncer as *LUCA1* (*HYAL1*), *LUCA2* (*HYAL2*) and *LUCA3* (*HYAL3*) [23]. This region was found to be deleted in many epithelial cancers and further analysis revealed the deleted region could be refined to a 120 kb interval that included *HYAL2* (*LUCA2*) [24]. However, *HYAL2* mutations were not identified in 40 lung cancer cell lines and overexpression of *HYAL2* in 4 chromosome 3p-deficient lung cancer cell lines did not alter cell proliferation or apoptosis rates [24]. To date, none of the hyaluronidase genes have been identified as tumour-susceptibility genes (TSG) [25]

More recently, two additional genes, *CEMIP* (originally *KIAA1199*) and *CEMIP2* (most commonly known as *TMEM2*), which are not homologous to the previously described bee venom hyaluronidase, were found to be involved in HA degradation in humans and other species [26,27]. Though considerable evidence demonstrates that both bind to HA and facilitate the depolymerization of HA, it is still unclear as to whether they are true hyaluronidases as their mechanism of action is not yet fully understood [26,28,29,30,31]. These proteins contain a unique G8 domain which is comprised of eight conserved Gly residues and five β-strand pairs. PKHD1 is the only other protein found to have a G8 domain; mutations in this protein cause an autosomal recessive form of polycystic kidney disease [32]. CEMIP and TMEM2 proteins both have a G8 domain, GG domains, and PbH1 domains. Though highly similar in sequence (48% amino acid identity), differences include a transmembrane domain in TMEM2, while CEMIP has an N-terminal signal peptide sequence.

The characterization of humans, mice, and zebrafish with a deficiency of a protein involved in HA degradation has been critical in directing investigations of how HA is turned over and its impact on biological processes. The discovery, phenotypes and molecular basis of human and animal deficiencies of each HA-degrading enzyme and how this has influenced our understanding of the field will be reviewed.

## 2. Human Disorders of Hyaluronidase Deficiency

Very few individuals with hyaluronidase deficiency have been identified to date, but the impact of the characterization of their phenotypes in advancing our understanding of HA turnover has almost certainly been undervalued in the field. The phenotypes of human patients have often motivated further studies of an animal model or even driven a re-assessment of our understanding of HA metabolism. The phenotypes of these patients will be described below with a brief overview of the basic features of the affected gene/protein.

### 2.1. HYAL1 Deficiency Is the Cause of MPS IX

#### 2.1.1. Phenotype of HYAL1 Deficiency

A genetic form of hyaluronidase deficiency was first recognized in a 14-year-old female proband who presented at age 7 with a soft tissue mass over her left ankle [33]. Periarticular soft tissue masses involving several other joints developed over the next two years; at times, these masses were swollen and painful. Joint effusions, soft tissues masses around multiple joints, and erosion of the acetabula were evident on imaging. The proband also exhibited short stature (below the fifth percentile at 14 years of age), a flattened nasal bridge, bifid uvula and a submucosal cleft palate. There was no evidence of neurological or visceral involvement. These findings were consistent with hyaluronidase deficiency, but a robust description of the clinical phenotype awaited characterization of additional individuals with HYAL1 deficiency.

Histological and biochemical studies provided strong evidence that the proband had an autosomal recessive form of hyaluronidase deficiency. Histological examination of biopsies from two distantly located periarticular masses revealed abundant vacuolated histiocytes and smaller numbers of vacuolated fibroblasts were identified in the skin. These vacuolated cells could be stained with Alcian blue and colloidal iron which detect mucopolysaccharides, and pre-treatment with hyaluronidase decreased the staining. Using a biochemical assay, no hyaluronidase activity was detected in the proband’s plasma and approximately 50% of normal activity was found in the parents’ plasma. Further, HA levels in the proband’s plasma were elevated 30–90-fold compared to the levels in normal plasma. Taken together with the knowledge that HA is abundant in synovial fluid, this disorder was identified as a genetic deficiency of serum hyaluronidase. In 1999, this deficiency, termed MPS type IX or MPS9 (OMIM 601492) was found to be caused by variants in the gene encoding HYAL1 [18]. MPS IX is referred to by some as Natowicz syndrome after the discovering physician.

Additional individuals with HYAL1-deficiency were identified through homozygosity mapping in a consanguineous Middle Eastern family presenting with familial juvenile idiopathic arthritis [34]. The proband was a 13-year-old male with right knee pain and swelling. On imaging, proliferative synovitis with a small effusion was observed in the right knee, and later joint ultrasonography indicated that there were abnormalities and synovitis in all imaged joints, even those of the hand. Evaluation with MRI imaging of two affected siblings revealed effusions in multiple joints. Synovial biopsies from these three subjects demonstrated vacuolated histiocytes, consistent with the presence of accumulating HA. The findings in this family were isolated to the joints, with no short stature or involvement of other tissues.

MPS9 is the rarest and mildest of the known MPSs. It is likely that some individuals with MPS9 are not recognized because of their similarity to other disorders such as juvenile idiopathic arthritis and pigmented villonodular synovitis. Possible differential indicators of HYAL1 deficiency could be multiple affected family members or steroid-resistant symptoms. However, in analysis of 108 juvenile idiopathic arthritis patients in Turkey, none were found to have serum hyaluronidase deficiency [35], reaffirming the rarity of this condition. Based on the phenotypes of individuals identified with HYAL1 deficiency at this time, the most characteristic findings are soft tissue masses involving multiple joints and evidence of joint effusions on MRI imaging.

#### 2.1.2. The Molecular Basis of MPS9

All individuals affected with MPS9 were found to have a complete deficiency of serum hyaluronidase activity using zymography [18,34]. In the first proband identified [33], sequencing of the most broadly and highly expressed hyaluronidase-encoding genes, *HYAL1* and *HYAL2*, revealed variants only in *HYAL1*; NM_033159.4:c. 751_787delinsTTCCGTGTGGCCCG (p.Val251fs) and c.802G>A (p.Glu268Lys). Sequencing of DNA from the three siblings described in 2.1.1 revealed a c.104delT(p.Val35Alafs23) variant in *HYAL1* that was homozygous in all of the affected subjects. Truncated proteins are unlikely to be produced because the premature stop codons are expected to result in nonsense-mediated decay of the transcripts. When first identified, the Glu268Lys variant was proposed to cause complete HYAL1 deficiency because the Glu268 is conserved in all related hyaluronidases and when this residue was substituted with Gln in either human PH20 or bee venom hyaluronidase, it disrupted enzyme activity [36]. However, structural studies showed Glu268 was distant from the active site [37] and most likely impacted HYAL1 by disrupting electrostatic interactions leading to protein instability. Based on the crystal structure, the side chain of Glu268 forms a hydrogen bond with Asn229 and a salt bridge with Arg271, residues that are also conserved in human hyaluronidases. An in silico study also supported the deleterious effect of Glu268Lys [38].

#### 2.1.3. HYAL1 Gene and Protein

An acid-active serum hyaluronidase was first separated from the neutrally-active testicular hyaluronidase (now known as PH20) in 1967 [39]. This activity was later purified from human serum and cloning of the HYAL1 cDNA revealed it to be a 435 amino acid protein [40] which migrated on a native gel as 57 kDa in serum and 45 kDa in urine [41]. The complete absence of serum activity in HYAL1-deficient individuals suggested that it was the only active hyaluronidase in serum. HYAL1 has three domains including a signal sequence, a catalytic domain, an epidermal growth factor domain [42], and three N-glycosylation sites [43] and has been crystallized as a distorted 8-stranded α/β barrel [44]. HYAL1’s acidic pH optimum and broad distribution in tissues suggested that it was involved in the lysosomal degradation of HA but the strongest evidence to support this was the lysosomal accumulation of HA in MPS9 patient histiocytes and, to a lesser extent, fibroblasts [33]. HYAL1 was not elevated in the serum of patients deficient in the enzymes responsible for generating the mannose-6-phosphate targeting signal on lysosomal enzymes, suggesting it primarily reached the lysosome by a phosphomannosyl-independent pathway [45]. In a series of elegant in vitro and in vivo studies, HYAL1 was shown to be trafficked to lysosomes through a precursor form that was secreted and then taken up by a mannose receptor pathway for transport to the lysosome [46,47]. The HYAL1 precursor was taken up by the mouse liver in vivo [47] and subsequent studies showed that it is then processed in the lysosome [46,48]. The uptake of HYAL1 is also important for HA internalization [49].

**Figure 2:**
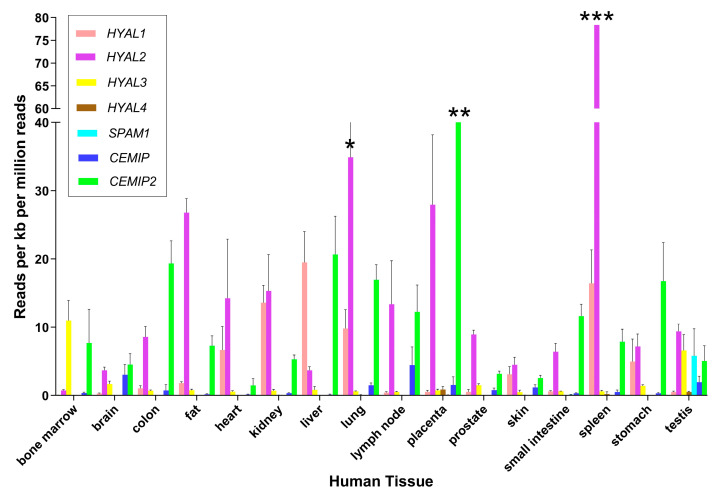
Expression of genes encoding HA-degrading proteins in human tissues. Estimates of gene expression were determined by RNA-Seq in a previous study [51]. Mean ± standard deviation is shown for the major tissues where RNA-Seq data were available from at least 3 different biological replicates. Results are graphed for *HYAL1*, *HYAL2*, *HYAL3*, *HYAL4*, *SPAM1*, *CEMIP1* and *CEMIP2* using GraphPad Software Version 10 (Boston, MA, USA). To facilitate viewing, some error bars are not fully graphed and are indicated by * (SD = 9.81), ** (SD = 1.84), and *** (SD = 21.8).

The absence of visceral HA accumulation in MPS9 patients despite the high expression of HYAL1 in the liver, spleen and kidney was unexpected, and suggested that other enzymes might be compensating for HYAL1 deficiency in these tissues (Figure 2). Other hyaluronidases were already speculated to play a role in HA catabolism because of the low levels of HYAL1 expression detected in the brain. The accumulation of HA primarily in histiocytes of the joints of HYAL1-deficient subjects suggested that macrophage cells were primarily responsible for HA turnover in this tissue. Further, the absence of accumulation in other tissues indicated that either HA turnover was higher in the joints than other tissues or that the levels of compensating enzymes in the joints were insufficient. Based on these findings, other HA-degrading enzymes were presumed to be the primary mediators of catabolism in some tissues (i.e., the brain) and could compensate for HYAL1 deficiency in most tissues. The relative contribution of exoglycosidases β-hexosaminidase and β-glucuronidase to lysosomal degradation of HA was unknown [11,50] and their significance is described in the context of HYAL1 deficiency, in Section 3.

### 2.2. HYAL2 Deficiency Causes Syndromic Cleft Lip and/or Palate

#### 2.2.1. Phenotype of HYAL2 Deficiency

Variants in *HYAL2* were first identified as the putative cause of syndromic cleft lip and/or palate using homozygosity mapping of an extended Amish pedigree with five affected individuals, and subsequently confirmed in a Saudi Arabian family [52,53]. Soon, ten additional individuals of various ethnicities were identified that were either homozygous or compound heterozygous for *HYAL2* variants [54]. Some of these affected individuals had more severe clinical presentations than the original subjects, with death occurring within the first year of life in 3 of 10 cases. Based on the 17 affected individuals that had been identified, a full review of their clinical features was performed [54]. Among these individuals, cleft lip and/or palate was present in 10 of 17, and heart abnormalities in 12 of 17, individuals. Although only partial clinical information was available for some subjects, shared findings were evident. Of the subjects studied, 13 of 14 and 14 of 16 had a broad and flattened nasal bridge and hypertelorism, respectively; the craniofacial features of these individuals were strikingly similar. Other common features were external ear abnormalities (11/14), micrognathia (9/14), single palmar crease (9/13), pectus excavatum (7/16), myopia (11/11), and hearing loss (7/16). The range of cardiac abnormalities was broad, with many ventricular septal defects. Three individuals exhibited tetralogy of Fallot and one previously described subject had three atria (*cor triatriatum sinister*) [53]. For a full review of the unique findings in smaller numbers of subjects, please see [54]. These studies clearly showed an important role for HYAL2 during development and imply a need to regulate HYAL2 activity to control HA levels during development. Without tissues from humans with HYAL2-deficiency available for further studies, additional findings come from studies in HYAL2-deficient mice (described in Section 3).

#### 2.2.2. The Molecular Basis of HYAL2-Deficiency

A range of DNA variants in *HYAL2* have been identified, including the Amish *HYAL2* founder variant NM_003773.4: c.443A>G (p.Lys148Arg) [53], and a series of less-common variants, c.749C>T (p.Pro250Leu), c.829C>T (p.Arg277Cys), c.883C>T (p.Arg295*), c.194C>G (p.Ser65*), c.1273T>G (p.Phe425Val), c.611G>C (p.Gly204Ala), c.1271_1272delAC (p.His424Leufs*12), c.713T>G (p.Leu238Arg), c.1132C >T (p.Arg378Cys), and c.190G>A (p.Ala64Thr). These variants were found to be absent or uncommon in gnomAD although the c.443A>G variant found in Amish subjects had a frequency of 0.6% in the Anabaptist variant server [54]. Transient expression studies demonstrated that the variants that led to a premature stop codon (Ser65* and Arg295*) resulted in no detectable HYAL2 protein, probably because the *HYAL2* mRNA was subjected to nonsense-mediated decay. Most amino acid substitutions (Lys148Arg, Pro250Leu, Leu238Arg, Phe425Val, Arg277Cys) resulted in low levels of HYAL2 protein, consistent with a folding defect. Two of the variants (Gly204Ala and His424Leufs*12) resulted in apparently normal levels of expressed HYAL2 protein, but both microscopy and cleavage of the cell surface proteins from their GPI anchor indicated that they did not properly localize [53,54]. Two variants (Arg378Cys and Ala64Thr) did not have functional studies performed but were predicted to be deleterious, based on bioinformatic analyses.

#### 2.2.3. HYAL2 Gene and Protein

*HYAL2* was identified in 1998 as a homologue of other known hyaluronidases, including *PH20* (*SPAM1*) [19]. It was expressed in a broad range of human tissues, heart, placenta, lung, liver, skeletal muscle, kidney and pancreas, but not brain, thymus or leukocytes [17]. In the brain, *HYAL2* was found to be postnatally downregulated, consistent with a role during development [55].

The activity and localization of HYAL2 have been controversial [56]. In most cells, HYAL2 is a 52–60 kDa GPI-anchored protein [57] that can also serve as a receptor for the Jaagsiekte sheep retrovirus [56,57,58]. It was initially proposed to have weak activity toward high-molecular-mass HA at low pH to produce 20 kDa HA fragments [59], which led to models where cell surface HYAL2 interacts with an HA receptor to generate HA fragments that are internalized and degraded in the lysosome [60,61,62,63]. This model of HA degradation was received favorably and was supported with early studies of HA turnover in cartilage [64]. However, others have either not detected HYAL2 activity [65], found weak activity associated with a soluble form [66], or found evidence of regulated HYAL2 activity [67]. The range of developmental abnormalities observed in subjects deficient in HYAL2 are consistent with it being involved in the regulated degradation of HA to initiate or terminate specific signaling events during development. In addition to cell surface localization, HYAL2 is localized to α-granules in platelets and becomes surface-expressed when platelets are activated [68]. Mouse models have allowed more extensive characterization of HYAL2′s role in tissues.

### 2.3. HYAL3 Deficiency and Oligospermia

#### 2.3.1. Phenotype of HYAL3 Deficiency

*HYAL3* is broadly, albeit weakly, expressed in somatic tissues but has high expression in the bone marrow and testis [17]. *HYAL3* variants were reported in a single study as the autosomal recessive cause of male infertility associated with oligospermia [69]. Although several variants were identified in *HYAL3*, the absence of functional studies, follow-up studies or clear discussions relating to how these variants impact phenotype, makes a confirmatory publication important to validate these findings. Studies in mice with HYAL3 deficiency described in Section 3 may be of limited value because rodents have additional hyaluronidases expressed in sperm.

#### 2.3.2. The Molecular Basis of HYAL3 Deficiency

Sequencing of the *HYAL3* coding region was carried out for 11 subjects from Pakistan with male infertility, 7 due to oligospermia and 4 with secondary infertility [69]. Several variants were reported as stop codons that, instead, are likely missense mutations including R286X, where a review of the data presented in the paper indicates this variant is, in fact, NM_003549.4: c. 857G>A; (p.R286H). Other amino acid substitutions were reported in subjects with secondary infertility, including p.K168S, p.K168T, p.H113X, p.E164N, p. P162X and p.F157X, but additional studies are needed to assess the significance of these findings.

#### 2.3.3. HYAL3 Gene and Protein

HYAL3’s low level and restricted expression has led to limited studies of its function. It was detected in human sperm on Western blot analysis as 47 and 55 kDa bands [70], but most other analyses have described changes in *HYAL3* at the mRNA level in different pathological conditions.

### 2.4. CEMIP Deficiency

#### 2.4.1. Phenotype of CEMIP Deficiency

CEMIP (CEll Migration-Inducing Protein) also known as KIAA1199 or HYBID (hyaluronan-binding protein) was identified as a cause of non-syndromic hearing loss in four Japanese families during a screen of 52 genes that are specifically or preferentially expressed in the inner ear [71]. *CEMIP* is on human chromosome 15q25 and had not previously been identified as a locus involved in hearing loss. Some cases appeared to be autosomal recessive, while in others the inheritance pattern was unclear because only one variant allele was detected. The hearing-loss phenotype in these patients was highly variable, with differing ages of onset and severities of hearing loss [71]. The characterization of additional hearing-loss subjects with CEMIP variants is needed to further assess CEMIP’s role in hearing loss [72].

#### 2.4.2. The Molecular Basis of CEMIP Deficiency

Three different missense substitutions in *CEMIP*, NM_001293298.2: c.559C>T (p.R187C), c.560G>A (p.R187H) and c.2347C>T (p.H783Y) were identified; two (R187C and R187H) were found in familial cases of hearing loss and one (H783Y) was found in a sporadic case of hearing loss [71]. These residues are highly conserved in CEMIP and related family members. To assess the impact of these missense substitutions on function, the changes were introduced into a cDNA and expressed in COS-7 cells. The R187C and R187H mutations resulted in cytoplasmic localization, similar to wild-type CEMIP, while H783Y, resulted in an irregular cytoplasmic pattern and changes in cell morphology [71]. Subsequent localization studies have largely demonstrated that CEMIP is a secreted protein [28,73,74,75,76]. Later functional studies indicated that only the R187C and R187H substitutions reduced HA-degrading activity associated with CEMIP [26], providing additional evidence that they cause hearing loss. Of note, R187 is located at the N-terminal GG domain of CEMIP (amino acids 180–284), which is a lectin domain with carbohydrate-binding activity [77].

#### 2.4.3. CEMIP Gene and Protein

CEMIP was originally associated with hearing loss because of its high expression in cells of the developing inner ear of mice [71,78]. CEMIP expression has subsequently been found in a broad range of human tissues, with highest expression in the central nervous system, placenta, lung, and testis, with little or no expression in kidney, skeletal muscle, liver, or peripheral blood [79,80]. Many studies have now demonstrated that CEMIP expression is high in migrating cells and in multiple different cancer types, mostly of epithelial origin, with overexpression in many of these cancers associated with poor clinical outcome [81,82]. In addition to being a secreted protein, CEMIP has also been localized to the ER and Golgi [28,74,83], which is consistent with a secreted protein, in endosomes, [26,84], and potentially in the nucleus of a subset of colorectal cancers [85]. CEMIP’s role in HA degradation was identified during a screen for proteins upregulated by histamine and downregulated by TGF-β1 and was shown to be independent of CD44, HYAL1, and HYAL2, as well as the caveolin pathway [26]. CEMIP specifically binds HA [26] and leads to its degradation in the endosomes of fibroblasts, followed by secretion of HA fragments into the extracellular space.

## 3. Murine Models of Hyaluronidase Deficiency

Mice deficient in HYAL1, HYAL2, HYAL3, SPAM1/HYAL6, HYAL5, CEMIP, or TMEM2 have been vital to further our understanding of the role or potential role of these genes in HA turnover and/or disease. There is currently no mouse model for HYAL4 deficiency. HYAL4’s expression is limited to placental and skeletal muscle in humans [17], and biochemical studies have indicated that it specifically degrades chondroitin and not HA [86].

### 3.1. HYAL1 Deficiency in the Mouse

The relatively mild phenotype resulting from HYAL1 deficiency in humans was recapitulated in the *Hyal1−/−* mouse. These mice appeared grossly normal and had a normal life span [87]. Osteoarthritis was detected in the knee joints by reduced Safranin O staining, indicating a loss of proteoglycans that was evident at 3 months of age and progressively worsened with age. By 20 months of age, a complete loss of articular cartilage and other bone abnormalities in the joint were evident. In these joints, HA was increased in the matrix surrounding and within the chondrocytes, although HA was not found to be grossly elevated in other tissues. Further studies of the bone phenotype in aged *Hyal1−/−* mice revealed a decrease in bone mineral density that was accompanied by increased HA and decreased mineralization by *Hyal1−/−* osteoblasts [88]. Comprehensive studies in the *Hyal1−/−* mouse later showed that HYAL1 was important in HA degradation in peripheral tissues but its greatest role was in the non-parenchymal cells of the liver [89]. An increase in HA in various tissues of *Hyal1−/−* mice has been shown, including the kidney cortex and outer medulla [90], and the stratum corneum of the skin [91]. In the ovary, no increase in HA was detected, but *Hyal1−/−* mice exhibited increased primordial follicles and prolonged fertility [92].

The mild phenotype in HYAL1-deficient patients and mice suggested other enzymes might be involved in degrading HA. It was already known from studies of subjects with Sandhoff disease, due to β-hexosaminidase A and B deficiency, that glycosaminoglycans accumulated in some tissues [93,94]. To determine the relative importance of β-hexosaminidase A and B in the degradation of HA, HA levels were compared in mice that were deficient in both HYAL1 and β-hexosaminidase A/B to those deficient in HYAL1 or β-hexosaminidase A/B alone [14]. These studies clearly demonstrated that the exoglycosidases, β-hexosaminidase A/B could compensate for HYAL1 deficiency, possibly explaining the relatively mild phenotype associated with HYAL1 deficiency. The greatest accumulations of HA were in the liver and lymph node, consistent with the important role that they play in HA uptake and turnover. Importantly, HA accumulation in the brain of β-hexosaminidase A/B-deficient mice was substantially elevated when HYAL1 was also deficient, indicating that HYAL1 plays an important role in HA turnover in the brain, despite its apparent low expression.

### 3.2. HYAL2 Deficiency in the Mouse

A deficiency of HYAL2 in mice was originally thought to be lethal because *Hyal2−/−* mice were not obtained from heterozygous pairings [59]. However, on an outbred background approximately 1/3 of *Hyal2−/−* mice survived [95,96], with most dying between P1 and P7, and some at ~E15.5 [53]. Mice that survived did not exhibit extensive tissue HA accumulation as one might expect if it were required for all constitutive HA turnover [95], but HA accumulation was present in all tissues and highest in skin, lymph node, and ovary [97]. These mice exhibited many features that resembled the affected patients, including a broad nose, widely spaced eyes [95], submucosal cleft palate, hearing loss, and cardiac anomalies [53]. Additional findings in the mice that were not reported in the humans with HYAL2 deficiency included elevated serum HA, Wormian bones in the frontal suture, decreased mineralization of the craniofacial bones, cervical vertebral abnormalities, thrombocytopenia with anemia, missing kidney, and premature death [53,95,98]. Humans with HYAL2 deficiency were found to have myopia and other eye abnormalities, as well as skeletal defects, which have not yet been described in the mouse [54].

Studies of surviving *Hyal2−/−* mice showed that cardiac dysfunction was typically the cause of premature death. These mice had expanded heart valves with accumulating HA, as well as cardiac hypertrophy [96]. Ultrasound imaging showed that 50% of surviving mice had *cor triatriatum sinister,* an anomaly that was also detected in a human with HYAL2 deficiency [99]. Interestingly, mice with the most severe cardiac dysfunction did not have *cor triatriatum*, but rather massively distended atria, atrial masses, and elevations in mesenchymal cells throughout the heart. The mice exhibited diastolic dysfunction, ultimately leading to heart failure between 12 and 25 weeks of age [100]. In addition to the craniofacial and cardiac phenotypes, approximately 50% of outbred *Hyal2−/−* mice had only one kidney. The early death of *Hyal2−/−* mice has prevented the full characterization of the phenotypes that are associated with HYAL2 deficiency, but differences in the eyes and developing skeleton may be present.

Using conditional *Hyal2* KO mice, HYAL2 was shown to be dispensable for normal kidney function, although there was HA accumulation in the kidney [90]. In mice with the conditional removal of *Hyal2* in the cartilage, HA was found to accumulate and was accompanied with osteoarthritic changes in the knee joint, suggesting HYAL2 is important in the maintenance of chondrocyte function [101].

Overall, the human and mouse phenotypes were remarkably similar when they were compared, both sharing craniofacial, skeletal and cardiac defects. These findings suggest that HYAL2 is required for the normal development primarily of tissues derived from neural crest cells, where HA is highly expressed during development. Based on the presence of high levels of HA in these tissues, it is likely that HYAL2 activity is regulated to remove HA at specific times to allow differentiation of cells. The presence of increased numbers of mesenchymal cells in the hearts of *Hyal2−/−* mice is consistent with a delay in inhibition of mesenchymal cell proliferation and induction of differentiation. Further characterization of the mechanisms driving the increased mesenchymal cells in the HYAL2-deficient mice will likely inform the human disorder. It would also be helpful to determine if the cardiac phenotype in the mouse benefits from therapy with circulating hyaluronidase; this could be beneficial for human patients if the cardiac problems are found to worsen with age.

### 3.3. HYAL3 Deficiency in the Mouse

Mice deficient in HYAL3 (*Hyal3−/−*) were not found to have any obvious morphological differences or evidence of HA accumulation [102]. Sperm from *Hyal3−/−* mice were found to have delayed cumulus penetration and reduced acrosomal exocytosis, although they were fully fertile [70]. In contrast, there was no impact on the fertility in female *Hyal3−/−* mice [92]. Interestingly, the overexpression of mouse HYAL3 protein in bovine hamster kidney cells resulted in increased HYAL1 activity, suggesting that HYAL3 increases HYAL1 activity [103].

### 3.4. SPAM1/HYAL5 and HYAL6 Deficiency in the Mouse

PH20 was known as a protein in the sperm head before it was identified as a homologue of the bee venom hyaluronidase (reviewed in [104]). Mice that are deficient in SPAM1 (PH20/HYAL7) are fertile and have a 55 kDa protein that is active as a hyaluronidase [21]. This activity was found to be encoded by a new rodent-specific hyaluronidase, *Hyal5*, which is located close to *Hyal6 (HYAL6P* in humans) but is expressed only in the testis. Mice that are deficient in either SPAM1 or HYAL5 remain fertile [21,105]. However, mice deficient in both SPAM1 and HYAL5, i.e., *Spam1−/− Hyal5−/−*, had reduced fertility with fewer offspring than wild-type mice and an accumulation of sperm on the outside of the cumulus oocyte complex [106]. To determine if the testis-specific HYAL6 (HYAL6P pseudogene in humans) could contribute to fertilization, *Hyal6−/−* mice were created [107]. HYAL6 does not appear to contribute to hyaluronidase activity in the sperm and its absence does not affect fertility or cumulus–oocyte complex dispersal.

### 3.5. CEMIP Deficiency in the Mouse

Work by separate groups led to two different targeting strategies for the deletion of CEMIP in which either the entire 5′ UTR-containing exon1 is deleted (ΔE1), or to a 14-bp region in exon 4 is deleted [108,109]. In both models, mice with a deficiency in CEMIP (*Cemip−/−*) appeared grossly normal and viable, although in initial studies by Shimoda et al. the number of *Cemip−/−* offspring was lower than expected [108]. Similarly, both models demonstrated that CEMIP is highly expressed in normal bone and CEMIP deficiency impacts bone growth, though with different findings. Shimoda et al. found that CEMIP-deficient mice showed a delay in endochondral ossification and reduced angiogenesis, which is believed to be the cause of a small decrease in long-bone length [108]. However, Chen et al. showed that *Cemip−/−* mice had increased formation of osteoblasts which increased bone mass and strength, enhanced bone formation, and accelerated healing, suggesting that CEMIP normally functions as a regulator of osteoblast differentiation [109]. Finally, in a mechanically-induced knee osteoarthritis model, cartilage destruction and osteophyte formation were reduced in CEMIP ΔE1 mice, suggesting a role for HA degradation by CEMIP in osteoarthritis progression [110].

HA is one of the primary components of the ECM in both the dermis and brain [4]. As such, several studies have looked at the impact of CEMIP deficiency in the brain and dermis using the CEMIP ΔE1 mouse [111,112,113]. In the brain, HA is important for nervous system development and maintenance of normal function [114]. The hippocampus is a particularly HA-rich region of the brain [115]. CEMIP has an important role in the brain, as in its absence high-molecular-mass HA accumulated in the hippocampus of *Cemip−/−* mice, which was accompanied by spatial memory impairment [111]. Interestingly, these mice did not exhibit hearing impairment [111]. It is unknown if *Cemip −/−* mice in studies by Chen et al. exhibit a similar lack of hearing impairment [109]. Further studies to better understand the cognitive impairment found in *Cemip −/−* mice showed decreased neurons in the dentate gyrus and decreased dendritic spine density that was accompanied by HA accumulation, suggesting HA degradation by CEMIP is important for synaptic formation [112]. Finally, in dermal studies, *Cemip−/−* mice exhibited resistance to Staphylococcus aureus infection, which was thought to be conferred by decreased degradation of high molecular mass HA and a resultant increase in inflammation and antimicrobial activity [113]. Our understanding of CEMIP is still evolving and further studies of mice with CEMIP-deficiency will be essential to determine how it relates to other enzymes involved in HA degradation.

### 3.6. TMEM2 Deficiency in Zebrafish and Mouse Models

TMEM2 was first recognized as a potential hyaluronidase, based on its homology with CEMIP, as well as the finding of HA accumulation in zebrafish *tmem2* mutants [116,117]. Additional studies in zebrafish found that TMEM2 plays a major role in cardiac development through regulation of myocardial and endocardial morphogenesis and atrioventricular valve differentiation, as well as skeletal muscle morphogenesis through regulation of cell–matrix interactions [117,118,119]. TMEM2 is now speculated to be the major hyaluronidase involved in extracellular HA degradation [31] and has been demonstrated to show intrinsic hyaluronidase activity [120]. Consistent with this, mice with a tamoxifen-induced global TMEM2 deficiency had greatly elevated levels of HA in the serum, liver, lung, kidney, skin and bone marrow in just three weeks of treatment, suggesting that TMEM2 had an important role in systemic HA turnover [121]. Further, TMEM2 was localized to endothelial cells of the lymph nodes and liver sinusoids, important sites for systemic HA catabolism, all suggestive of TMEM2 playing an important role in systemic HA degradation. In separate studies, the same group found that *Tmem2* expression is high in the neural tube, frontonasal region, branchial arches and heart, as well as other neural crest-derived tissues. To study TMEM2’s role in the development of neural crest-derived tissues, Wnt1-Cre conditional *Tmem2−/−* mice were generated. These mice displayed a range of craniofacial, cardiac and brain malformations that were accompanied by accumulating HA [122]. They show that *Tmem2−/−* neural crest cells undergo increased apoptosis and death, resulting in hypoplasia of neural crest-derived tissues and demonstrating an important role for TMEM2 in normal neural crest development.

## 4. Summary

The existence in vertebrates of multiple proteins involved in the degradation of HA likely reflects the importance of its turnover in vertebrates. Each of the proteins described herein can contribute to HA degradation and yet, except for HYAL1 and the exoglycosidases, which are clearly involved in the constitutive degradation of HA in the lysosome, we still lack clarity on how, where and when they function. The developmental abnormalities associated with HYAL2 or TMEM2 deficiencies indicate they are likely regulated proteins required during development. However, their roles in constitutive degradation are less defined. It is interesting that HYAL2 and CEMIP2 show remarkably similar patterns of expression in mammalian cells and mice, and their deficiency exhibits similar phenotypes. This raises the question of whether the two may functionally interact with each other. Similarly, it is also interesting to consider whether HYAL1 and CEMIP may function together. Despite HYAL1’s known role in the lysosome, it is largely a secreted protein that is subsequently internalized, raising the possibility that it may have a relationship with CEMIP, which is also secreted. Future studies that explore these relationships will help to clarify the functions of these proteins and whether they have any shared roles in HA catabolism.
